# Stars, stripes and scripts: Innovations and challenges in US pharmacy

**DOI:** 10.1016/j.fhj.2024.100201

**Published:** 2024-12-12

**Authors:** Roshan Tasgaonkar

**Affiliations:** OMS-IV at the West Virginia School of Osteopathic Medicine (WVSOM), 400 Lee St, Lewisburg, WV 24901, USA

## Abstract

Pharmacy practice in the United States has evolved, transitioning from simple dispensing to complex compounding and medication management. Pharmacists play important roles across many sectors, including clinical care, pharmaceutical industry, insurance companies, and public health. Pharmacists optimize therapy, improve patient outcomes, and reduce healthcare costs. However, the profession faces challenges such as burnout, understaffing and declining insurance reimbursements, particularly in independent pharmacies. Pharmacy Benefit Managers (PBMs) have exacerbated these issues, controlling drug prices and reducing pharmacy profitability through opaque pricing and monopolistic practices. Recent legislative efforts in states like New York have aimed to curb PBM influence, however issues persist, and nationwide legislation is lacking. The future of pharmacy depends on addressing these challenges through unified lobbying and proactive reform. Despite these hurdles, scope of practice and innovation within the field continues to grow, offering hope for a revitalized pharmacy landscape.

Pharmacy practice in the USA has come a long way. From the days of Bill and Fill’ dispensing and compounding to modern practice, which includes complex compounding and novel medication delivery such as multi-dose blister packing, medication synchronisation, immunising, advanced disease state education, collaborative drug therapy management (CDTM) and more. The field has experienced a proliferation of novel ideas and talent.

The scope of the US pharmacist has correspondingly grown over the decades, resulting in a broad presence across various healthcare roles. The board-certified pharmacotherapy specialist (BCPS) specialises in medical disciplines, providing valuable skills and input to clinical rounding teams in US hospitals. By optimising therapy and dosing, and preventing adverse drug reactions, they enhance patient safety and reduce healthcare expenditures. Pharmacists are also found in the pharmaceutical industry where they contribute to strategy, acquisitions, medication development, clinical trials and more. Pharmacists interface with physicians and allied providers as medical science liaisons (MSLs) helping to educate on new medications and clinical trial data. Pharmacists work in insurance companies such as CVS Caremark, Blue Cross Blue Shield and Humana.^1^ Here, they interface with pharmacies and hospitals to adjudicate medication claims and ‘prior authorisations’ that require insurance approval for high-value medications. They also engage in auditing. In essence, pharmacists work full spectrum across healthcare.

COVID-19 underscored the vital importance of the community pharmacist. Pharmacies served as a haven for medications and vaccinations and a forum where an anxious public could interact with healthcare professionals. In those early days, US pharmacists immunised hundreds of millions of people and educated them about COVID-19.^2^ They served as a front-line weapon against disease, accessible to the public at large without appointment.

Chronic problems persist, however. Retail pharmacists overwhelmingly report burnout, being overworked and understaffed. They are expected to fill anywhere from 200 to 1,000+ prescriptions with a maximum of two technicians, while responding to an endless flood of patient and provider calls and faxes. All this while ensuring safety, efficacy and accuracy of dispensed medications and often skipping lunch. This, combined with excessive corporate bureaucracy and top-down micromanagement, leads to frustration, errors and dissatisfaction. CVS and Walgreens (owner of Boots), two of the largest retail chains, recently instituted a mandatory 30-minute lunch break, seen as a small victory for the retail pharmacist.^3^

Declining insurance reimbursements represent another chronic pressure. Independent pharmacies, traditionally ‘mom-and-pop’ neighbourhood institutions, have suffered the most. Independents lack the purchasing power and leverage of big chain pharmacies and cannot force better wholesaler pricing or insurance reimbursement rates. This places an unfair pressure, resulting in historic rates of store closure in this segment. A recent report from the state of Washington revealed that 83 pharmacies closed in the last 18-month period. This was partly explained by an increase in theft; however, the greatest blame was placed on pharmacy benefit managers (PBMs). The CEO of the Washington State Pharmacy Association, Jenny Arnold, said: ‘If a pharmacy decides not to take that (PBM) contract, they can lose a third of their business overnight. But it’s stuck between a rock and a hard place because they lose money on the contract, (or) they lose a third of their patients. It’s really a lose/lose situation for pharmacies.’^4^ The journal of the Missouri State Medical Association further adds: ‘PBMs use their market leverage to increase their profits, not reduce costs for consumers. Incredibly, PBMs own their own pharmacies. This ownership creates huge conflicts of interest, hurts competition, and distorts pricing.’^5^ (Fig. 1).^10^

**Fig. 1:**
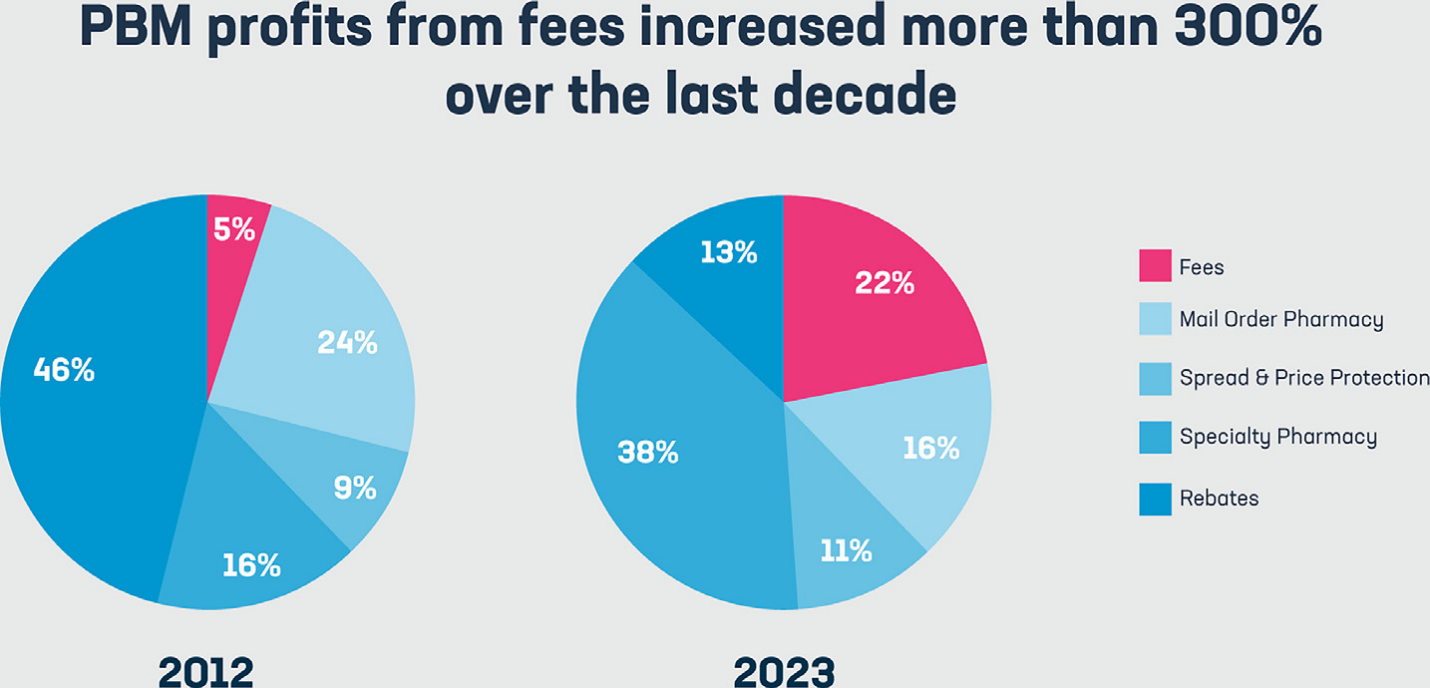
Pie charts showing breakdown of pharmacy benefit manager (PBM) profits, including from fees, which has increased substantially from 2012 to 2023.^10^

The relatively recent growth of PBMs or ‘middlemen’ insurance companies is loathed in the pharmacy community. Originally designed to negotiate prices with drug companies and control spiralling costs, their scope has increased to include claims processing and managing pharmacy networks (Fig. 2). They wield disproportionate power in the pharmacy value chain but provide dubious value. They have paradoxically increased costs and reduced reimbursements, with opaque ‘spread pricing’ that differs from pharmacy to pharmacy and is designed to extract maximum profit. PBMs have enriched themselves at the expense of pharmacies, limited outside competition with their in-house pharmacies and compromising care to vulnerable communities. These rapacious ‘middlemen’ are partly responsible for the decline in retail pharmacy, especially among independents.

**Fig. 2:**
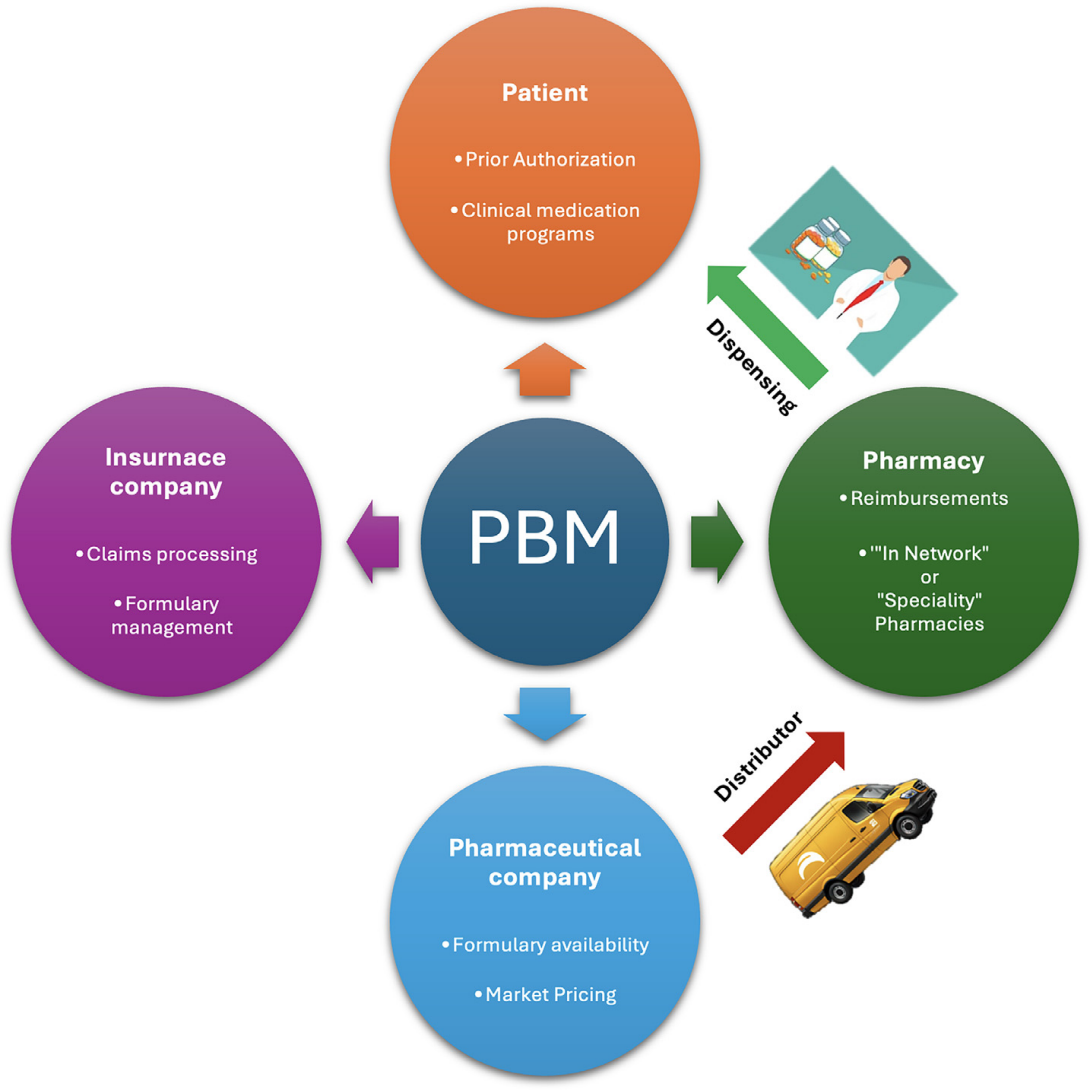
Schematic of pharmacy benefit manager (PBM) involvement in US healthcare. PBMs are centrally positioned to interface with healthcare participants.

There has been pushback. Multiple states, including Washington, are drafting legislation to defend against unregulated PBM growth. New York (NY) and other states have enacted legislation that restricts PBM participation with the state’s most socio-economically stressed (Medicaid) patients. The NYRx programme returns NY to a fee-for-service model, allowing the state to pay pharmacies directly for dispensed medications. This provides NY with ‘full visibility’ into prescription drug costs, centralises and leverages negotiation power and provides a single drug formulary statewide. Medicaid patients may now access any pharmacy in NY instead of being ‘restricted’ from ‘out of network’ pharmacies due to arbitrary PBM rules.^6^

Although welcome, these changes are too late. Scores of independents have been shuttered nationwide and will never serve their communities again. The pharmacist community has complained about PBMs for over a decade before meaningful legislation was on the table. The reactionary pace of progress reflects another chronic pressure on the profession – the inadequacy of pharmacy lobbying power at the national level. Pharmacists’ interests are not being defended by a single coherent voice. There exist multiple organisations with overlapping goals that result in ineffectuality. Physicians, in contrast, rely on the American Medical Association (AMA), which has one of the largest lobbying budgets in the USA. The AMA vigorously and proactively defends physician interests at multiple levels.^7^ The same political nous and organisational potency is sorely needed in pharmacy. Pharmacists should be defending against negative forces long before they disrupt the profession.

When comparing pharmacy systems in America and the UK, there are obvious differences in size and scale. Healthcare budgets are predictably larger in America (which accounts for over half of the world’s total) (Fig. 3),^8^ but size is not always king. There is great fragmentation in the myriad insurance companies, PBMs and incompatible non-communicating software systems. In addition, the absence of universal healthcare means that there is no single formulary which the federal government (purchaser) can negotiate prices on. Individual states and businesses negotiate pricing directly with manufacturers and suppliers, which is opaque. The UK, in contrast, benefits from a single software interface, or digital spine,^9^ connecting healthcare organisations nationwide and enhancing healthcare delivery. The UK also utilises its national purchasing power to negotiate better pricing, resulting in lower medication costs across the board. This model allows for greater transparency in costs, more efficient software integration and more value for dollars spent.

**Fig. 3:**
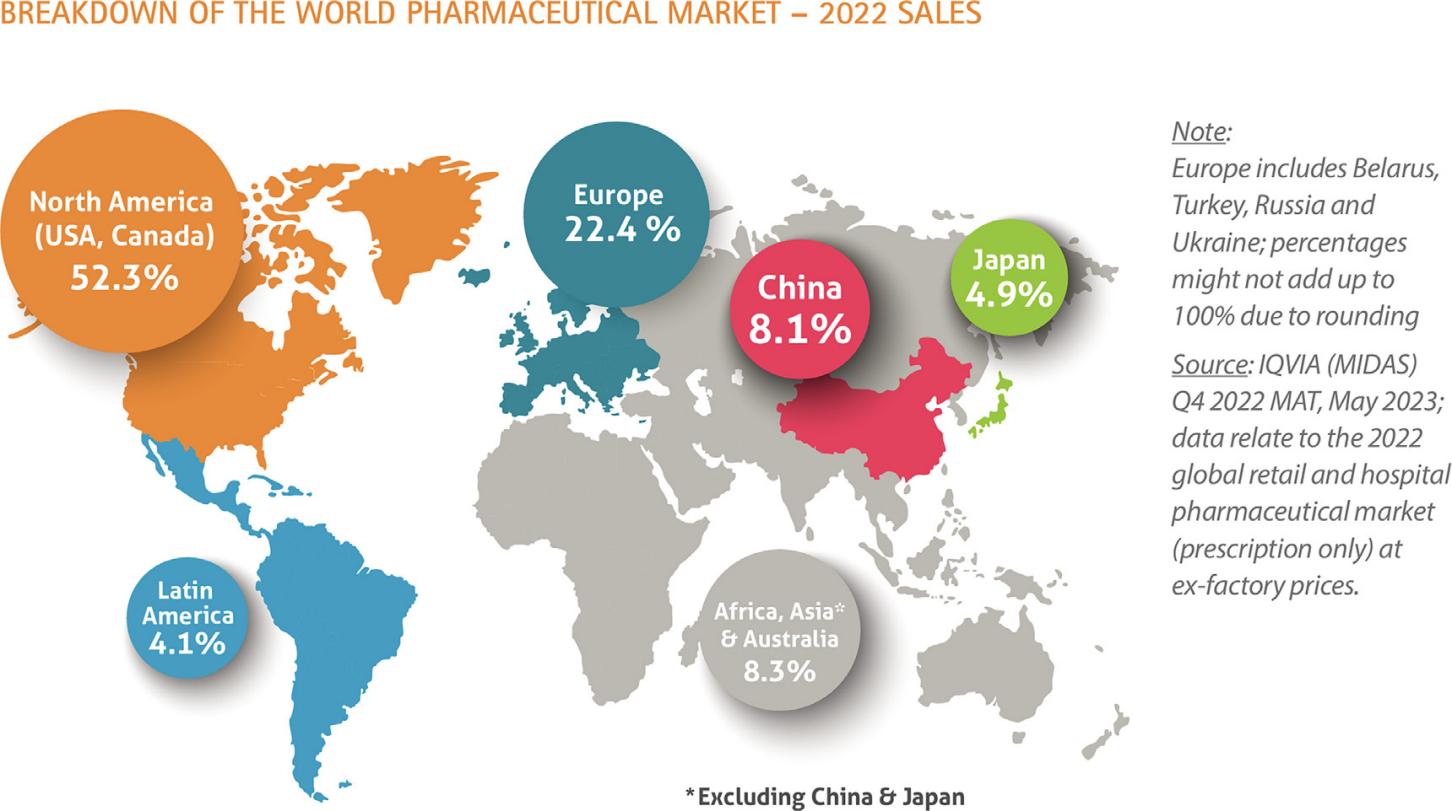
Map of global pharmaceutical sales in 2022, broken down by geographic region, and as a percentage of total worldwide sales.^8^

My journey has taken me from pharmacy school to independent pharmacy practice in NY, where I worked as pharmacist in charge of a large team for 8 years. I then transitioned to school and am completing my final year of medical school in West Virginia. My journey has also shown me a side of healthcare from a student physician perspective. I see parallels such as reimbursement pressure which unduly guides care. However, I see my future career as complementary to pharmacy. There are opportunities to work in the pharmaceutical industry as a physician and I see myself revisiting pharmacy in some capacity, perhaps as part of pharmacy management. Why did I make the switch? Ultimately, I wanted greater clinical impact on patient care and medicine allowed me to grow my knowledge base to improve this care.

There is great hope in the future of American pharmacy. Scope of practice increases steadily, and pharmacies continue to innovate and provide novel services. The US pharmacy student has more job options than ever before, in a dynamic and evolving field. However, the systemic pressures buffeting the profession must be addressed proactively and with a unified lobbying effort. PBMs have been exposed as counterproductive to the common effort and a return to fee-for-service model, as implemented in NY, is recommended. Reimbursement pressure must also be addressed so that pharmacy work is remunerated fairly. I anticipate re-engaging with pharmacy in my future. I love the profession and wish to remain connected in my work as a future physician. I dream of a revitalised field, incorporating new skillsets, unleashing new potential and earning new stars and stripes.

## Funding

This research did not receive any specific grant from funding agencies in the public, commercial, or not-for-profit sectors.

## CRediT authorship contribution statement

**Roshan Tasgaonkar:** Visualization, Writing – review & editing.

## Declaration of competing interest

The authors declare that they have no known competing financial interests or personal relationships that could have appeared to influence the work reported in this paper.

## References

[1] Kissell C. Largest health insurance companies. 2024. Forbes Magazine; 2024 [Accessed June 12, 2024]. Available from: https://www.forbes.com/advisor/health-insurance/largest-health-insurance-companies/.

[2] Grabenstein JD. (2022). Essential services: quantifying the contributions of America's pharmacists in COVID-19 clinical interventions. J Am Pharma Assoc.

[3] D'Onofrio M. CVS pharmacies close daily for 30-minute lunch break.2022 [Accessed June 12, 2024]. Available from: https://www.axios.com/local/philadelphia/2022/02/28/cvs-pharmacies-close-lunch-break-hours.

[4] Jones J. 83 pharmacies closed across Washington state in 18 months. KIRO 7 News Seattle; 2024 [Accessed June 12, 2024]. Available from: https://www.kiro7.com/news/jesse-jones/jesse-jones-83-pharmacies-closed-across-washington-state-18-months/LA6B26CL4VF7BKSHRPBBS2CEUQ/?utm_source=MarketingCloud&utm_medium=email&utm_campaign=PT%2BDaily%2BJune%2B7&utm_content=PT%2BDaily%2BJune%2B7.

[5] Gale A. (2023).

[6] Transition of the pharmacy benefit from managed care (MC) to Medicaid NYRx Pharmacy Program. NYS DOH; 2023 [Accessed June 12, 2024]. Available from: https://www.health.ny.gov/health_care/medicaid/redesign/mrt2/pharmacy_transition/pharmacy_transition_faq.htm.

[7] American Medical Assn lobbying profile. 2023 [Accessed June 12, 2024]. Available from: https://www.opensecrets.org/federal-lobbying/clients/summary?cycle=2023&id=D000000068.

[8] European Federation of Pharmaceutical Industries and Associations; 2023 [Accessed August 14, 2024]. Available from: https://efpia.eu/media/rm4kzdlx/the-pharmaceutical-industry-in-figures-2023.pdf.

[9] SpineNHS; 2024 [Accessed August 14, 2024]. Available from: https://digital.nhs.uk/services/spine.

[10] Ubl SJ. New analysis shows pbms use fees as a profit center 2023 [Accessed August 14, 2024]. Available from: https://phrma.org/Blog/New-analysis-shows-PBMs-use-fees-as-a-profit-center.

